# Identification and Characterization of *Verticillium nonalfalfae*-Responsive MicroRNAs in the Roots of Resistant and Susceptible Hop Cultivars

**DOI:** 10.3390/plants10091883

**Published:** 2021-09-11

**Authors:** Urban Kunej, Jernej Jakše, Sebastjan Radišek, Nataša Štajner

**Affiliations:** 1 Department of Agronomy, Biotechnical Faculty, University of Ljubljana, 1000 Ljubljana, Slovenia; urban.kunej@bf.uni-lj.si (U.K.); jernej.jakse@bf.uni-lj.si (J.J.); 2 Plant Protection Department, Slovenian Institute of Hop Research and Brewing, 3310 Žalec, Slovenia; sebastjan.radisek@ihps.si

**Keywords:** *Humulus lupulus*, *Verticillium nonalfalfae*, biotic stress, microRNA, high-throughput sequencing

## Abstract

MicroRNAs are 21- to 24-nucleotide-long, non-coding RNA molecules that regulate gene expression at the post-transcriptional level. They can modulate various biological processes, including plant response and resistance to fungal pathogens. Hops are grown for use in the brewing industry and, recently, also for the pharmaceutical industry. Severe Verticillium wilt caused by the phytopathogenic fungus *Verticillium nonalfalfae*, is the main factor in yield loss in many crops, including hops (*Humulus lupulus* L.). In our study, we identified 56 known and 43 novel miRNAs and their expression patterns in the roots of susceptible and resistant hop cultivars after inoculation with *V. nonalfalfae*. In response to inoculation with *V. nonalfalfae*, we found five known and two novel miRNAs that are differentially expressed in the susceptible cultivar and six known miRNAs in the resistant cultivar. Differentially expressed miRNAs target 49 transcripts involved in protein localization and pigment synthesis in the susceptible cultivar, whereas they are involved in transcription factor regulation and hormone signalling in the resistant cultivar. The results of our study suggest that the susceptible and resistant hop cultivars respond differently to *V. nonalfalfae* inoculation at the miRNA level and that miRNAs may contribute to the successful defence of the resistant cultivar.

## 1. Introduction

Hops (*Humulus lupulus* L.) are traditionally cultivated for use in the brewing industry as an essential ingredient that provides flavour and acts as a stabilizer or preserver of the beer [1]. In recent years, the bioactive compounds of hops have also become increasingly attractive for use in the pharmaceutical industry [2,3]. One of the main limiting factors in hop production are fungal diseases, especially those caused by the soil-borne plant pathogenic fungus *Verticillium nonalfalfae* (formerly known as *Verticillium albo-atrum*) [4]. The symptoms of Verticillium wilt in hops caused by *V. nonalfalfae* vary depending on the pathogenicity of the fungal strain and the sensitivity of the cultivar. Susceptible hop cultivars can suffer from severe symptoms (e.g., leaf chlorosis and necrosis) and also complete dieback of rootstock caused by a highly virulent strain of *V. nonalfalfae* [5].

To prevent infections with various phytopathogens, plants have evolved multi-tiered defence mechanisms. The first layer of defence is represented by pattern recognition receptors (PRRs) present at the cell membrane surface that recognize conserved pathogen-associated molecular patterns (PAMPs). This phenomenon is called PAMP-triggered immunity (PTI) or basal defence. PTI comprises both physical and chemical defence responses, e.g., the deposition of lignin-like compounds in the cell wall, the production of reactive oxygen species (ROS) and the activation of signalling cascades that modulate gene expression. Successful pathogens can counteract the plant basal immune response by deploying the effectors into the cytoplasm of plant cells to attenuate defence. Their presence in the cytoplasm is directly or indirectly detected by receptors, so-called resistance (R) proteins, or nucleotide-binding leucine-rich repeat (NB-LRR) proteins encoded by *R*-genes. Resistance mediated by *R*-genes is considered to be the second layer of defence and confers an enhanced type of defence, known as effector-triggered immunity (ETI) [6,7]. To achieve the best effectiveness of the defence response, all defence mechanisms must be well regulated. In this dynamic and complex network of gene regulatory pathways during the immune response, short non-coding RNAs, so-called microRNAs (miRNAs), play a pivotal role [8,9]. miRNAs are a class of small endogenous non-coding RNA molecules with a length of 21 to 24 nucleotides, which act as post-transcriptional regulators of gene expression and are thus involved in various biological processes [10]. Recently, Soto-Suarez et al. [11] demonstrated that miR396 mediates the PAMP-triggered immune response against necrotrophic and hemibiotrophic fungal pathogens in *Arabidopsis*. Navarro et al. [12] discovered that in *Arabidopsis* plants treated with PAMP (Flagellin fragment 22), miR393 was upregulated and, as a result, the expression of F-box auxin receptors was silenced, leading to suppression of the auxin signalling pathway and enhanced PTI. The upregulation of miR393 was also detected in soybean in response to infection with the pathogenic fungus *Phytophthora sojae*. Moreover, miR393-knockdown soybean plants show increased susceptibility to infection [13]. However, miRNAs other than miR393 can modulate the auxin signalling pathway in various pathogen infections and are involved in plant immune response. For example, miR160 regulates auxin response factors (ARFs) in potato and, thereby, indirectly affects the expression of genes that modulate salicylic acid–auxin cross-talk, which is associated with local defence and systemic acquired resistance to *Phytophthora infestans* [14]. NB-LRR proteins, products of *R*-genes that mediate ETI in plants, are targeted by several miRNAs, such as miR2118 in *Medicago truncatula* [15] and in cotton infected with *Verticillium dahliae* [16], by gma-miR1510a/b, which contributes to the resistance to *Phytophthora sojae* [17], and by ptc-MIR482, ptc-MIR1447 and ptc-MIR1448 in *Populus trichocarpa* [18,19].

Although there is increasing evidence that miRNAs play an important role in the regulation of gene expression during the immune response in plants, there is a scarcity of information on miRNA-mediated gene silencing during the pathogenesis of fungal diseases, such as Verticillium wilt, in various crops. In the resistant hop cultivar Wye Target, a single quantitative trait locus (QTL) has been identified, which explains 26% of the phenotypic variance for Verticillium wilt resistance [20], and potential resistance gene analogue-expressed sequence tag (RGA-EST) markers have been developed [21]. A well-studied example of Verticillium wilt resistance to date relates to the tomato’s *Ve1* gene, which codes for a leucine-rich repeat (LRR) receptor-like protein that confers the resistance to a strain of *V. dahlia* race 1 or *V. nonalfalfae* [22,23,24,25]. *Ve1* orthologue was also characterized in hops and it is suggested that it provides the resistance to *V. dahliae* strain 1 by detecting an effector protein Ave1 [26]. In a proteomic study, Mandelc et al. [27] observed an accumulation of defence-related proteins, such as chitinase, β-glucanase, thaumatin-like protein, peroxidase and germin-like proteins in the compatible interactions between *V. nonalfalfae* and hops, while such a response was not detected in the incompatible interactions. Similarly, an increased expression of genes is involved in innate immunity; the jasmonic acid pathway and wounding was observed in the roots and shoots of the susceptible hop cultivar [28], while Cregeen et al. [29] observed the increased expression of genes involved in ubiquitination (SKP1), vesicle trafficking (cdc48), protein degradation (puromycin-sensitive aminopeptidase), protein–protein interactions (syntaxin and Fk506), transport (acyl-CoA-binding protein) and morphogenesis (furry protein) in the resistant cultivar and decreased expression in the susceptible cultivar.

In the present work, we characterized miRNAs in hops and identified *V. nonalfalfae*-responsive miRNAs in the roots of the susceptible and resistant hop cultivars. Furthermore, we used an in silico approach to predict transcripts targeted by *V. nonalfalfae*-responsive miRNAs and discussed their potential role in the defence response to *V. nonalfalfae* in susceptible or resistant hop cultivars based on their interactions within a local protein–protein interaction network cluster and their gene ontology.

## 2. Results

### 2.1. High-Throughput Sequencing of H. lupulus miRNAs

To investigate the miRNA response in roots of susceptible and resistant hop cultivars after inoculation with *V. nonalfalfae*, small RNA libraries were constructed from three control and three inoculated root samples of both hop cultivars. A total of 90,355,033 reads were obtained, ranging from 5,222,013 to 10,059,037 reads per library with a mean read length of 14 bp to 20 bp. After processing the raw sequencing data, we obtained from 1,771,295 to 4,636,681 sequencing reads with a mean read length of 18 bp to 22 bp per library. Processed reads from each library were aligned against the hop draft genome [30,31] disallowing mismatches (Table 1).

### 2.2. Known and Novel miRNAs Identified in Hop Root Tissue

**Figure 1:**
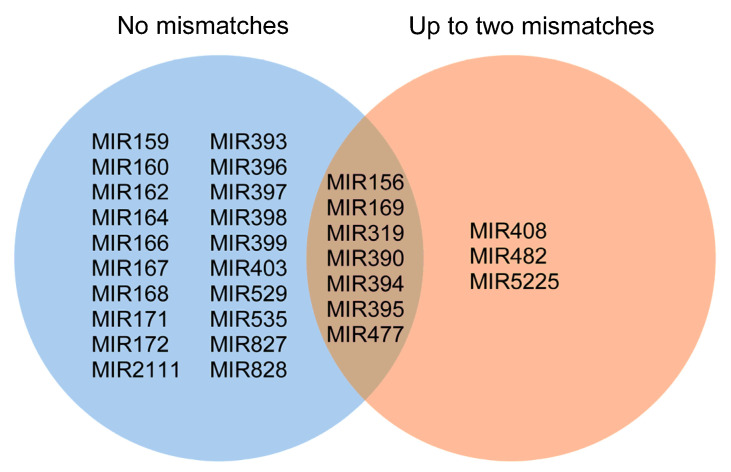
Families of miRNAs to which belong predicted hop miRNAs that align with known miRNAs deposited in miRBase. Hop miRNA families with members that align without mismatches are in the blue circle (20 miRNA families) and those that align with up to two mismatches are in the orange circle (3 miRNA families). Seven families comprise members that align perfectly or with up to two mismatches.

Reads perfectly aligned against the hop genome were subjected to miRNA prediction analysis using the miR-PREFeR pipeline [32], which predicted 2591 *MIR* loci (miRNA candidates) and their mature, precursor and star miRNA sequences. Of the 2591 miRNA candidates, 120 aligned with known miRNAs from miRBase [33] and 2471 miRNA candidates were novel. Of the 120 known miRNA candidates identified, 100 miRNA candidates encoded 44 different mature miRNAs (miR) that perfectly aligned with known miRNAs that belong to 27 different miRNA families (MIR) from miRBase [33], and 20 miRNA candidates encoded 12 different mature miRNAs that aligned with up to two mismatches against known miRNAs belonging to 10 different miRNA families (Figure 1, Table S1). Aligning-predicted miRNA precursor sequences (pre-miRNAs) against RNA sequences deposited in the RNAcentral database [34] did not result in additional annotations. The names of known miRNAs identified in *H. lupulus* were assigned based on the criteria and conventions for miRNA naming (Supplementary Table S1) [33,35]. Identified known miRNA families were not evenly represented in the number of members. The most represented families were MIR169 with five members; MIR156 and MIR477 with four members; MIR160, MIR167, MIR171 and MIR319 with three members; MIR159, MIR172, MIR390, MIR393, MIR394, MIR395, MIR399 and MIR482 with two members and the remaining 15 families were represented by a single member (Table S1). Additionally, the same mature miRNAs of the same family derive from a different number of precursor miRNAs or *MIR* loci. For example, MIR169 members derive from two (hlu-miR169m, hlu-miR169n) to six (hlu-miR169g, hlu-miR169h, hlu-miR169i, hlu-miR169j, hlu-miR169k, hlu-miR169l) different miRNA precursors and are altogether coded by 16 *MIR* loci.

Seven miRNA families comprise miRNAs that align perfectly or with up to two mismatches with known miRNAs of the corresponding miRNA family deposited in miRBase. Hlu-miR169o–p, hlu-miR319g–i, hlu-miR390b–c, hlu-miR394c–d, hlu-miR395c, hlu-miR477e, hlu-miR482a–b and hlu-miR482c aligned with one mismatch against known miRNAs from miRBase and are also very similar to other members of their family. With two mismatches, the following miRNAs were aligned against known miRNAs from miRBase; hlu-miR156g, hlu-miR477c–d, hlu-miR5225 and hlu-miR408a–b (Figure 1).

The most abundant miRNAs in the susceptible cultivar were hlu-miR482a–b and hlu-miR482c with, on average, 26,633 and 33,356 normalized read counts, respectively, followed by hlu-miR159a–b with, on average, 23,689 normalized read counts. Aforementioned miRNA families were also the most abundant in the resistant cultivar; hlu-miR482a–b had on average 30,089, hlu-miR482c had 29,664 and hlu-miR159a–b had 17,212 normalized read counts.

Of the 2471 predicted novel miRNA candidates, those with at least 100 reads mapped to the predicted mature miRNA and at least one read to the corresponding star miRNA, were considered as highly reliable predictions. Thus, we obtained 43 reliable predictions of novel mature miRNAs derived from 152 miRNA precursors. The names of novel mature miRNAs identified in *H. lupulus* were given as “miRNA-” followed by a consecutive number of the prediction but are not in order because unreliable predictions have been removed (Supplementary Table S2). Where novel predicted mature miRNAs were derived from different precursor sequences, these miRNAs were assigned two or more slash-separated precursor names, e.g., miRNA-363/miRNA-1427.

Using CD-HIT-EST [36] to cluster precursor sequences of novel miRNAs and pre-miRNAs from miRBase, we identified 89.01% similarity between the predicted precursor of miRNA-405 and miR156v (MI0022992) from *Malus domestica*. Furthermore, the mature sequence of miRNA-405 aligned with three mismatches (e-value: 0.36) against csi-miR156g-3p (MIMAT0048860) from miRBase. Based on this evidence, we assigned the novel miRNA-405 to the MIR156 family and named it as hlu-miR156h.

Additionally, clustering precursor sequences showed 81.37% similarity between one novel miRNA derived from four different precursors (miRNA-665, miRNA-2226, miRNA-2474, miRNA-2537) and miR395j from *P. trichocarpa* (MI0002324). These miRNA precursors were assigned to the MIR395 family, whereas the mature miRNA was treated as novel. The remaining novel miRNA precursors were clustered into thirty-three clusters, which were treated as novel miRNA families (Table S2).

### 2.3. Differentially Expressed miRNAs between V. nonalfalfae-Inoculated and Control Samples

The differential expression analysis of known and novel predicted mature miRNAs between inoculated and control samples was performed separately for each cultivar. miRNAs having at least 100 read counts in the susceptible or resistant cultivar were included in the differential expression analysis. miRNAs with the FDR corrected *p*-value ≤ 0.1 were considered as significantly differentially expressed. The results of differential expression analysis indicate that different members of the same miRNA family differ significantly in expression levels (Supplementary Table S1). Furthermore, except for hlu-miR477f and hlu-miR159c–d, which show similar expression patterns in the susceptible and resistant cultivars inoculated with *V. nonalfalfae*, other DE miRNAs differ between the two cultivars.

**Figure 2:**
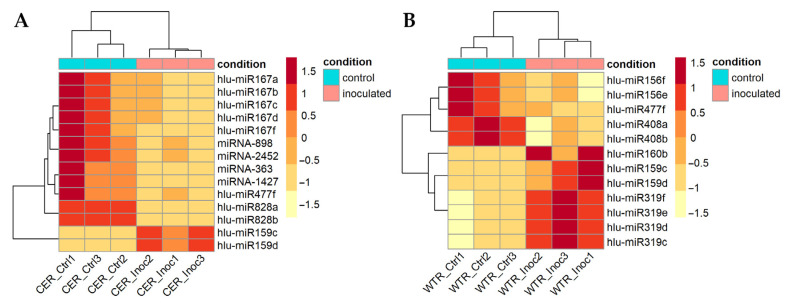
Heat map of differentially expressed miRNAs between *V. nonalfalfae*-inoculated (Inoc) and control (Ctrl) root samples (three biological replicates) of (**A**) the susceptible cultivar Celeia (CER) and (**B**) the resistant cultivar Wye Target (WTR). The colour scale represents the z-score scaled by row of the normalized read counts. Clustering was performed using the Euclidean distance measure and complete clustering method.

In *V. nonalfalfae*-inoculated root samples of the susceptible cultivar Celeia, we identified seven differentially expressed miRNAs, five of which belong to four different miRNA families (MIR159, MIR828, MIR477 and MIR167) and two novel miRNAs (miRNA-363/miRNA1427 and miRNA-898/miRNA-2452) belong to two different novel miRNA families. A significant upregulation was detected for hlu-miR159c–d (log_2_FC = 1.2) and a significant downregulation for hlu-miR828a–b (log_2_FC = −3.5), hlu-miR477 (log_2_FC = −2.1) and two members of MIR167, i.e., hlu-miR167f (log_2_FC = −2.1) and hlu-miR167a–d (log_2_FC = −1.5). Additionally, a significant downregulation was detected for two novel miRNAs, namely miRNA-363/miRNA-1427 (log_2_FC = −2.6) and miRNA-898/miRNA-2452 (log_2_FC = −2.5) (Figure 2a and Table S3).

In *V. nonalfalfae*-inoculated root samples of the resistant cultivar Wye Target, we identified six known differentially expressed miRNAs that belong to six different miRNA families (MIR408, MIR477, MIR156, MIR160, MIR319 and MIR159). Three miRNAs were upregulated, i.e., hlu-miR160b (log_2_FC = 1.2), hlu-miR319c–f (log_2_FC = 0.9) and hlu-miR159c–d (log_2_FC = 1), while hlu-miR408a–b (log_2_FC = −1.4), hlu-miR477f (log_2_FC = −1.6) and hlu-miRR156e–f (log_2_FC = −0.65) were downregulated (Figure 2b and Table S4).

### 2.4. Differentially Expressed MiRNAs between Susceptible and Resistant Hop Cultivars

**Figure 3:**
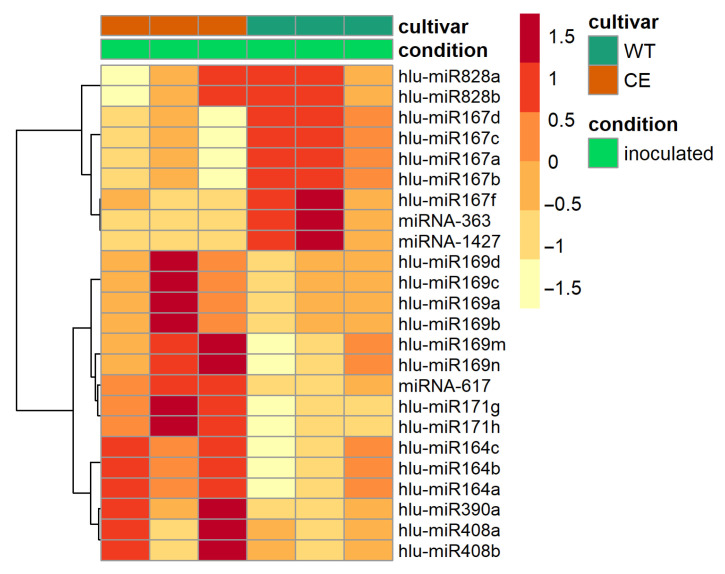
Heat map of differentially expressed miRNAs between the susceptible cultivar Celeia (CE) and the resistant cultivar Wye Target (WT) in response to inoculation with *V. nonalfalfae*. Clustering was performed using the Euclidean distance measure and Ward clustering method. The colour scale represents the z-score scaled by row of the control-normalized read counts.

Comparing miRNA response to *V. nonalfalfae* inoculation between susceptible and resistant hop cultivars, the resistant cultivar WT shows higher expression of hlu-miR167a–d and hlu-miR167f (log_2_FC = 1.3, respectively), hlu-miR828a–b (log_2_FC = 2.5) and a novel miRNA family containing miRNA-363 and miRNA-1427 (log_2_FC = 2.4), and are thus clustered together (Figure 3). On the other hand, hlu-miR390a (log_2_FC = −2.2), hlu-miR169a–d (log_2_FC = −2.2), hlu-miR169m–n (log_2_FC = −1.9), hlu-miR164a–c (log_2_FC = −1.4), hlu-miR408a–b (log_2_FC = −3.9), hlu-miR171g–h (log_2_FC = −1.3) and a novel miRNA-617 (log_2_FC = −6.3) shows lower expression in response to inoculation with *V. nonalfalfae* (Figure 3 and Table S5).

### 2.5. MiRNA Target Prediction, GO Analysis of miRNA Targets and Protein-Protein Interaction Network Analysis

In silico psRNATarget analysis [37] of miRNA target transcripts was performed for differentially expressed miRNAs of susceptible and resistant hop cultivars and revealed 49 transcripts that are potential targets (Table 2 and Table S8). For a single miRNA, psRNATarget identified from one to up to nine different target transcripts and one transcript is targeted by more than one DE miRNA (Table 2). Transcription factor GAMYB (W9QVM8) is targeted by two different miRNAs; hlu-miR159c–d, which is 1.28- and 0.95-fold (log_2_) upregulated in the susceptible and resistant cultivar, respectively, and by hlu-miR319c–f, which is 0.85-fold (log_2_) upregulated in the resistant hop cultivar. Most of the identified targets of DE miRNAs were found to encode transcription factors or transcriptional regulators. For example, auxin response factor (ARF) is targeted by hlu-miR160b, which is upregulated in the resistant cultivar. Moreover, some targets encode proteins involved in effector-triggered immunity, e.g., wall-associated receptor kinase (a target of novel miRNA-617) [38], or are involved in metabolic pathways (polyphenol oxidase; a target of novel miRNA-363/miRNA-1427) [39].

Gene ontology analysis (GO) showed that the targeted transcripts of the susceptible cultivar are enriched for twenty-one GO terms of biological processes with six targets that significantly contribute to enriched GO terms (significant targets), while seven enriched GO terms with eight significant targets were identified for molecular functions (Table 2 and Table S6). In the resistant cultivar, GO analysis revealed fifty-one enriched GO terms with five significant targets in the biological process ontology and six enriched GO terms with sixteen significant targets in the molecular function ontology (Table 2 and Table S7).

It is noteworthy that susceptible and resistant cultivars alter the expression of different miRNAs in response to inoculation with *V. nonalfalfae* and, therefore, the biological processes and molecular functions of their targets are expected to differ. In the susceptible cultivar, enriched biological processes of the DE miRNA targets include transportation and localization, i.e., protein retention in ER lumen, maintenance of protein localization, vesicle-mediated transport and cytosolic transport, and pigment biosynthetic process. Enriched molecular functions of the DE miRNA targets are ER retention sequence binding, signal sequence binding and catechol oxidase activity (Table S6). Targets of DE miRNAs in the resistant cultivar are involved in the auxin-activated signalling pathway, various regulatory processes, e.g., regulation of DNA-templated transcription, macromolecule biosynthetic process, nitrogen compound metabolic process and aromatic compound biosynthetic process, etc., and have various binding functions (Table S7).

**Figure 4:**
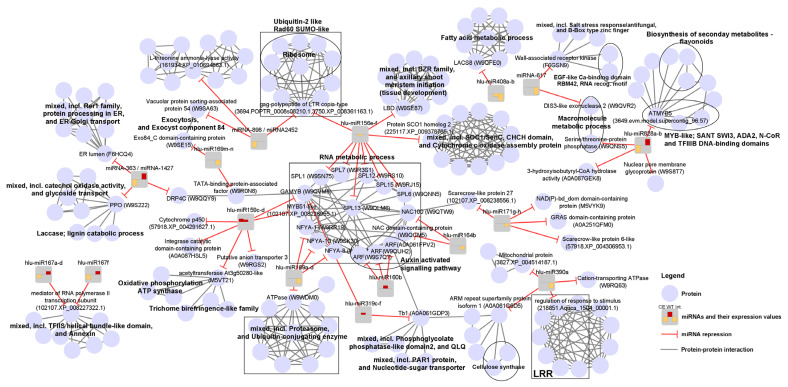
Protein–protein interaction network of miRNAs and their targets with interactors. Blue circles represent proteins and interactions between them (grey lines), and grey round rectangles with bar plot represent miRNAs repressing their targets (red lines with T-shaped arrowhead indicating target repression). The first and second bars of the miRNA bar plot represent miRNA expression levels (red, upregulated; orange, downregulated) determined by DESeq2 analysis for the sensitive hop cultivar Celeia (CE) and the resistant hop cultivar Wye Target (WT), respectively, upon inoculation with *V. nonalfalfae*. The third bar represents the interaction (Int.), defined as the difference between the condition effect for the resistant cultivar compared to the condition effect for the susceptible cultivar. Enriched terms of local network clusters obtained by the STRING Enrichment analysis are shown in bold. miRNA target names have either UniProt (Entry) or STRING identifier. ARF, Auxin response factor; NF-YA, Nuclear transcription factor Y subunit A; SPL, Squamosa promoter-binding-like protein; DRP4C, Dynamin-related protein 4C; LBD-LOB domain-containing protein; LRR, Leucine-rich repeat proteins; Tb1, Teosinte branched 1; LACS8, Long chain acyl-CoA synthetase 8; ER, endoplasmic reticulum.

The protein–protein interaction network of miRNA targets and their interactors comprises a total of 194 nodes (proteins or miRNAs) and 475 edges (connections), with each node connected to an average of 5.4 neighbours (interactors). Network analysis of protein–protein interactions of miRNA targets and their interactors revealed that the local network clusters comprising the targets of downregulated miRNAs in the susceptible hop cultivar are enriched in terms related to the TFIIS helical bundle-like domain, annexin, catechol oxidase activity, glycoside transport, laccase, Rer1 family, protein processing in ER, ER–Golgi transport, MYB-like domain, macromolecule metabolic process and biosynthesis of secondary metabolites (Figure 4). On the other hand, the upregulated miRNA local network clusters are enriched in terms related to cytochrome, transport, oxidative phosphorylation, ATP synthase, and trichome birefringence-like family. The latter three terms are also enriched in the upregulated miRNA local network clusters of the resistant hop cultivar. In addition, in the resistant hop cultivar, local network clusters with miRNA targets of upregulated miRNAs are enriched in terms related to the RNA metabolic process, auxin-activated signalling pathway, phosphoglycolate phosphatase-like, QLQ domain, PAR1 protein, and nucleotide sugar transporter (Figure 4). Local network clusters with targets of downregulated miRNAs in the resistant cultivar are enriched for terms related to the RNA metabolic process, BZR family, axillary shoot meristem initiation (tissue development) and fatty acid metabolic process. Moreover, local network clusters with significantly lower miRNA expression in the resistant cultivar compared to the susceptible hop cultivar after *V. nonalfalfae* inoculation, showed enrichment in terms related to leucine-rich repeat (Figure 4).

## 3. Discussion

We sequenced 12 sRNA libraries from three control and three *V. nonalfalfae*-inoculated roots of the susceptible cultivar Celeia or the resistant cultivar Wye Target. Using the miR-PREFeR miRNA prediction pipeline, we identified 56 mature miRNAs belonging to 30 known plant miRNA families and 35 novel miRNA families represented by a different number of members.

In our study, a significant upregulation in response to inoculation with *V. nonalfalfae* was observed for hlu-miR159c–d in both hop cultivars. Similarly, as reported, miR159 was upregulated in *P. beijingensis* inoculated with *D. gregaria* [19], *P. trichocarpa* inoculated with *Botryosphaeria dothidea* [40] and in *Triticum aestivum* during *Puccinia graminis* f.sp. *tritici* infection [41]. The main role of the MIR159 family is the regulation of *GAMYB* or *GAMYB-like* transcription factors that possess highly conserved binding sites for miR159 [42]. Previous studies have shown that miR159 represses primary root growth by inhibiting *MYB33*, *MYB65* and *MYB101* [43]. In silico miRNA target prediction revealed that hlu-miR159c–d (upregulated in both cultivars) and hlu-miR319c–f (upregulated in the resistant cultivar) bind to transcripts of *GAMYB* but also have other distinct targets. Moreover, hlu-miR319c–f targets transcripts of the hops’ teosinte branched 1 (*tb1*) (A0A061GDP3), which belongs to the TCP transcription factor family. While both miRNAs regulate MYB TFs in *Arabidopsis*, miR319 acts predominantly on transcription factors of the TCP family and, to a lesser extent, on the expression of *MYB* since the expression levels and domain of miR319 limit its regulation of *MYB*, while the sequence of miR159 prevents binding to *TCP* transcripts [44,45]. The latter is also observed in our study as we did not detect the binding site of miR159 within *TCP* transcripts. Teosinte branched 1 acts as a negative regulator of lateral branching [46,47], therefore its downregulation by upregulated hlu-miR319c-f in the resistant hop cultivar may lead to the secondary growth and branching of roots that may in turn help to sustain the vitality of the resistant cultivar during infection with *V. nonalfalfae*.

A significant downregulation was observed in our study for hlu-miR156e–f in the resistant hop cultivar, and in silico target prediction showed that it targets transcripts of six different Squamosa promoter-binding-like protein genes (*SPL*), SCO1 homolog 2 and LATERAL ORGAN BOUNDARIES (LOB) domain-containing protein (*LBD*). The interaction between hops’ miR156 and *SPB15* transcripts was previously confirmed by 5′ RLM-RACE analysis [48]. Furthermore, Bhogale et al. [49] validated the interaction between miR156 and *StSPL3*, *StSPL6*, *StSPL9*, *StSPL13* in *Solanum tuberosum* ssp. *andigena* and observed that miR156 can be transported through the plants via the phloem. Proteins from the SPB family are thought to be transcriptional activators and have roles in leaf development, vegetative phase change, flower and fruit development, plant architecture, shoot maturation, gibberellin signalling and response to fungal toxin [50,51].

Another interesting target of hlu-miR156e-f is the transcript of the LOB domain-containing protein (W9SE87). The latter protein family is involved in secondary growth and the development of xylem and phloem tissue [52] through a positive feedback loop that promotes the expression of the NAC domain-containing protein 30 (*NAC030*)/vascular-related NAC domain protein7 (*VND7*), which regulates genes associated with the differentiation of tracheary elements in *Arabidopsis*, e.g., genes involved in secondary wall biosynthesis, cell wall modifications such as xylan accumulation and programmed cell death [53,54]. Vascular-related NAC domain7 TF plays an important role in the response to infection with *V. longisporum* in *Arabidopsis*, as it induces de novo formation of functional xylem elements from bundle sheath cells, which subsequently leads to vein clearing and xylem hyperplasia within the vasculature of the roots, as well as to enhanced drought tolerance [55]. This may suggest that downregulation of hlu-miR156e-f and the resulting expression of *SPL* and *LBD* in the resistant hop cultivar modulates root development and vascular tissue processes in roots, which could contribute to a successful defence response after inoculation with *V. nonalfalfae*.

In response to inoculation with *V. nonalfalfae*, the resistant cultivar showed significantly lower expression of hlu-miR164b, which targets transcripts of NAC domain-containing proteins. The hlu-miR164b cleavage site within the transcript of the hops’ NAC domain-containing protein was confirmed by 5′ RLM-RACE analysis performed by Mishra et al. [48]. Hu et al. [56] observed a significant decrease of ghr-miR164 in the response of cotton plants to infection with *V. dahliae*. Additionally, the researchers showed that ghr-miR164 directly cleaves the mRNA of *GhNAC100*, and silencing of ghr-miR164 leads to increased *GhNAC100* expression, which in turn increases plant resistance to the fungus. The decrease of miR164 was also observed in *Oryza sativa* upon infection with *Magnaporthe oryzae* strain Guy11, and rice plants with the dysfunctional miR164a/OsNAC60 regulatory module developed a significant susceptibility to infection with Guy11 [57]. Auxin-induced expression of miR164 in wild-type *Arabidopsis* plants resulted in decreased levels of the *NAC1* transcripts and reduced lateral root emergence. Additionally, *Arabidopsis mir164a* and *mir164b* mutants that express less miR164 show higher expression of *NAC1* and have abundant lateral roots. This evidence may indicate that the auxin-miR164-*NAC1* regulation provides a homeostatic mechanism that modulates auxin signalling during lateral root development [58].

Hormone signalling pathways modulate plant responses to biotic stress [59] and may be involved in a trade-off between primary growth and response mechanisms of the resistant hop cultivar during Verticillium wilt pathogenesis. Hlu-miR160b, which is significantly upregulated in inoculated compared to control root samples of the resistant hop cultivar, is predicted to target transcripts of auxin response factors (*ARF*), DNA-binding proteins that bind to a specific sequence in promoters of auxin-responsive genes [60]. Upregulation of miR160 and its regulation of *ARF*s has also been demonstrated during the pathogenesis of stem canker disease in *P. trichocarpa* [40] and in potato, where it targets *St*ARF10, which binds to the promoter in the *StGH3.6* gene, a mediator of salicylic acid–auxin cross-talk, and is thus associated with local defence and systemic acquired resistance to *P. infestans* [14]. In *A. thaliana*, miR160 controls root cap formation by regulating the expression of *ARF10* and *ARF16*. Disturbed miR160-directed regulation of *ARF16* resulted in reduced fertility and fewer lateral roots [61]. In addition, researchers observed defects in root growth in *Arabidopsis* plants expressing an miR160-resistant version of *ARF17* [62].

The downregulation of hlu-miR156e-f, and the upregulation of hlu-miR160b and hlu-miR319c-f in inoculated root samples compared to the controls of the resistant hop cultivar, and the significantly lower expression of hlu-miR164b in the resistant hop cultivar compared to the susceptible hop cultivar, may indicate the role of these miRNAs in modulating hormone signalling and the processes of root growth, branching and vascular tissue development in the resistant hop cultivar during infection with *V. nonalfalfae*.

Compared to the susceptible hop cultivar, the resistant cultivar showed significantly lower expression of MIR169 and, by in silico analysis of miRNA targets, we identified nuclear transcription factor Y (*NF-YA*) as a target of miR169. Similarly, Li et al. [63] observed a higher expression of miR169 in the susceptible *O. sativa* cultivar but not in the resistant cultivar when infected with *M. oryzae*. In rice, miR169 suppresses the expression of NF-YA genes and, thus, acts as a negative regulator in rice immunity against blast fungus *M. oryzae*, since the transgenic lines that overexpress miR169 become hypersusceptible to *M. oryzae* due to the reduced expression of defence-related genes [63]. A significantly lower expression of miR169 in the resistant hop cultivar might, thus, contribute to hop resistance. In addition, miR169 regulates *NF-YA2* and *NF-YA10* genes involved in the control of primary root growth [64], further suggesting that processes of root growth are pronounced in the resistant hop cultivar inoculated with *V. nonalfalfae*.

The resistant hop cultivar inoculated with *V. nonalfalfae* also showed significantly lower expression of hlu-miR390a compared to the susceptible hop cultivar. One of its targets is involved in the regulation of response to stimuli and encodes proteins with successive leucine-rich repeat motifs. This hop protein may belong to the class of Toll-like receptors that bind pathogen- and danger-associated molecular patterns [65] and may be involved in the defence response to *V. nonalfalfae* infection. Similarly, after inoculation, the resistant cultivar showed significantly lower expression of the novel miRNA-617 than the susceptible hop cultivar. The cleavage site of miRNA-617 was predicted in transcripts of wall-associated receptor kinase from the protein family of receptor-like kinases (RLK), which are involved in the recognition of pathogens and signal transduction during pathogen attack [38].

In the resistant cultivar, we observed a downregulation of hlu-miR408a–b and a significantly lower expression of the latter in response to inoculation with *V. nonalfalfae* compared to the susceptible hop cultivar. Yin et al. [66] observed an increased expression of miR408 in *Arabidopsis* plants that overexpressed the effector protein SR1-a of *Puccinia graminis* f. sp. *tritici* (Pgt), but the increase was not significantly higher in wheat leaves. In our study, the binding site of miR408 was found in transcripts of the long chain acyl-coenzyme synthetase 8 (*LACS8*) that is associated with fatty acid metabolic process, and *LACS1* and *LACS2* that are involved in cutin biosynthetic process. In *A. thaliana*, the long chain acyl-coenzyme synthetase activates C16 or C18 fatty acids, which represent a substrate for cutin and wax [67]. The downregulation of miR408 may modulate the biosynthetic pathways of cutin and wax, which could lead to the accumulation of these compounds in the roots of the resistant hop cultivar when inoculated with *V. nonalfalfae*. Moreover, Progar et al. [28] observed enriched biological processes related to cell wall biogenesis and cutin biosynthesis in the transcriptomic study of interactions between *V. nonalfalfae* and resistant hop cultivar Wye Target.

In the susceptible hop cultivar inoculated with *V. nonalfalfae*, we observed a significant downregulation of hlu-miR167a–d, hlu-miR167f and hlu-miR828a–b, and two novel miRNAs, i.e., miRNA-363/miRNA-1427 and miRNA-898/miRNA-2452. All aforementioned miRNAs, except miRNA-898/miRNA-2452, had significantly higher expression in the resistant cultivar in response to inoculation with *V. nonalfalfae*.

Recent studies showed that miR828 positively regulates phenylpropanoid biosynthesis either by direct cleavage of *MYB* transcripts or by cleaving the transcripts of trans-acting siRNA gene 4 (*TAS4*), which results in the production of ta-siRNAs that silence the expression of the *MYB* gene [68,69]. In our study, cleavage sites of hlu-miR828a–b were predicted in transcripts of serine/threonine-protein phosphatase, 3-hydroxyisobutyryl-CoA hydrolase and transcripts of genes encoding proteins with RNA pol II transcription regulator recruiting activity (*ATMYB5*). The latter protein contains DNA-binding domains of MYB-related proteins or SANT domain, and forms a complex with a basic helix-loop-helix protein and the WD40 protein family that is involved in regulation of the flavonoid pathway [70]. These transcripts significantly contribute to GO-enriched terms of molecular functions and biological processes related to peptide binding and pigment biosynthesis in the susceptible hop cultivar and are also present in the local network cluster enriched for biosynthesis of secondary metabolites.

In contrast to the resistant hop cultivar, in which we may observe pronounced miRNA-mediated regulation of processes related to root growth and vascular tissue development following inoculation with *V. nonalfalfae*, miRNA regulation in the susceptible hop cultivar mediates transcriptional reprogramming leading to changes in various metabolic processes.

Cleavage sites of the novel miRNA-363/miRNA-1427 were identified in transcripts of polyphenol oxidase, a protein from the family of the ER lumen retaining receptors and in dynamin-related protein 4C. In previous studies, novel miRNAs targeting polyphenol oxidase were identified in *P. trichocarpa* [18], *Salvia miltiorrhiza* [39], *Solanum tuberosum* [71] and *Vitis vinifera* [72]; however, they differ in sequence from miRNA-363/miRNA-1427 identified in our study. The other two potential targets, the protein of the ER lumen retaining receptor family and dynamin-related protein 4C, are both involved in cellular localization or transport and have contributed significantly to the enrichment of these biological processes and molecular functions. Additionally, novel miRNA miRNA-898/miRNA-2452 potentially targets transcripts of vacuolar protein sorting-associated protein, which is involved in protein transport between endosomes and the trans-Golgi network [73] and, in the susceptible hop cultivar, is significant in the GO biological process terms associated with transport.

## 4. Materials and Methods

### 4.1. Inoculation of Hop Plants

Hop plants of the susceptible cultivar Celeia and the resistant cultivar Wye Target were provided by Slovenian Institute for Hop Research and Brewing. Hop plants were vegetatively propagated as softwood cuttings in a greenhouse or as dormant cuttings from the rootstock. One-year-old rooted cuttings were used in the experiment. The plants were inoculated by root dipping method using the well-established protocol proposed by Flajsman et al. [74]. Briefly, the roots of 3 biological replicates of one-year-old rooted cuttings of each cultivar were immersed for 10 min in a suspension containing conidia of the highly virulent strain of *V. nonalfalfae* (PV1, isolate T2) (5 × 10^6^ conidia/mL), and the roots of 3 control plants of each cultivar were mock-inoculated using sterile water. Artificially inoculated and mock-inoculated (control) whole root tissues were sampled 24 h after inoculation. Roots were cut from stems at the first node, washed, freeze-dried with liquid nitrogen, and ground to a fine powder with mortars and pestles. Following grinding, the samples were stored at −80 °C until total RNA and small RNAs were isolated.

### 4.2. Small RNA Isolation, Library Construction and Sequencing

Small RNAs were isolated from 100 mg root tissue of both cultivars in three *V. nonalfalfae*-inoculated and three control replicates, using mirVana™ miRNA Isolation Kit (Waltham, MA, USA) according to manufacturer’s instructions for the enrichment of small RNAs. The quantity and quality of the small RNA-enriched sample and miRNA fraction were assessed with Agilent^®^ 2100 Bioanalyzer^®^ instrument (Agilent Technologies, Inc., Santa Clara, CA, USA) using Bioanalyzer Agilent^®^ Small RNA Kit, following the manufacturer’s instruction. Thus, we determined the input amount of small RNAs, to construct three control and three *V. nonalfalfae*-inoculated small RNA libraries for each cultivar. Small RNA libraries were constructed using the Ion Total RNA-Seq Kit v2 and Ion Xpress™ RNA-Seq Barcode 1–16 Kit following the manufacturer’s instructions. Briefly, adaptors were hybridized and ligated to small RNAs, and the reverse transcription was performed. Afterwards, purification and size-selection were performed using magnetic beads to obtain only miRNAs and other small RNAs to which barcodes were added through PCR amplification. The yield and size distribution of amplified cDNA libraries were assessed with Agilent^®^ 2100 Bioanalyzer^®^ instrument (Agilent Technologies, Inc., Santa Clara, CA, USA) and Agilent^®^ High Sensitivity DNA Kit to pool equimolar barcoded libraries of each cultivar separately. Three inoculated and three mock-inoculated barcoded libraries of susceptible or resistant cultivars were pooled in equimolar concentration and prepared for sequencing according to the manufacturer’s instructions, accompanying Ion PI™ Hi-Q™ OT2 200 Kit and Ion PI™ Hi-Q™ Sequencing 200 Kit. Both prepared samples were sequenced on the Ion Proton™ System (Waltham, MA, USA). The raw sequencing data were deposited to the SRA archive (BioProject ID PRJNA665133).

### 4.3. Prediction, Identification and Differential Expression Analysis of miRNAs in Hops

Prior to bioinformatics analysis, barcodes, adapters and low-quality raw sequence reads were removed using FastQC software (https://www.bioinformatics.babraham.ac.uk/projects/fastqc/; accessed on 30 August 2021) and high-quality sequencing reads were used for further analysis. Briefly, FASTA files containing sRNA-seq reads were pre-processed using scripts provided by miR-PREFeR pipeline [32] and the reads were aligned with Bowtie [75], disallowing mismatches, against hop draft genome sequences obtained from HopBase [30,31]. Afterwards, alignment files of processed RNA-Seq reads were used to predict hop miRNAs using a miR-PREFeR pipeline with parameters set according to criteria for plant miRNA annotation [76].

To identify known miRNA families in hops, predicted mature or precursor miRNA sequences were aligned with Bowtie2 [77] against mature or precursor sequences in the microRNA database (miRBase Release 22.1) [33]. Additionally, predicted precursor miRNA sequences were aligned against RNAcentral, a non-coding RNA sequence database [34].

Minimum folding energy (MFE) of secondary structures of predicted precursor miRNAs was calculated using RNAfold tool [78] and used to calculate adjusted minimal folding free energy (AMFE), which enables indirect comparison of MFEs among predicted known and novel pre-miRNAs [79].

sRNA counts of predicted mature miRNAs provided by miR-PREFeR output were subjected for differential expression analysis in R version 3.5.1 [80]. Count matrices containing read counts of control and inoculated samples were constructed for susceptible and resistant cultivars, respectively. Prior to differential expression analysis, predicted mature miRNAs with less than 100 read counts in control and inoculated samples were discarded. The differential expression analysis of predicted mature miRNAs was performed with DESeq2 [81]. Predicted miRNAs with FDR corrected *p*-value < 0.1 were treated as significantly differentially expressed between inoculated and control samples. Filtering according to the log_2_ fold change parameter was not applied because we wanted to detect low but significant changes in the expression of miRNAs.

To test whether the treatment effect differs across cultivars, the interaction term was added to the model in DESeq2 and the entire read-count matrix containing mature miRNAs with more than 200 read counts in all samples was used in the differential expression analysis. Predicted mature miRNAs with *p*-value ≤ 0.05 were considered as differentially expressed.

Furthermore, predicted precursors of novel miRNAs (novel pre-miRNAs) and known pre-miRNAs from miRBase were clustered using CD-HIT-EST [36] with a global sequence identity threshold 0.8. Predicted novel pre-miRNAs clustered with annotated pre-miRNAs were grouped into corresponding known miRNA families and predicted novel pre-miRNAs that did not show similarity were categorized as novel miRNA families.

### 4.4. In Silico Prediction of MiRNA Targets of Differentially Expressed MiRNAs, Gene Ontology and Protein–Protein Network Analyses

MicroRNA target analysis was performed on-line using psRNATarget Analysis Server (2017 Update) [37]. Mature miRNA sequences of differentially expressed miRNAs were used in in silico miRNA target prediction analysis. The targets of differentially expressed miRNA of susceptible and resistant hop cultivars were predicted in annotated hop transcriptome [82] with the following parameters: max expectation cut-off: 2.5, seed region: 2–13, number of mismatches allowed in seed region: 2, range of mismatch disable slicing: 9–11, HSP length for scoring: 19, penalty for GU pair: 0.5, penalty for other mismatches: 1.0, allowing bulge on target, penalty for opening gap: 2.0, penalty for extending gap: 0.5, weight for seed region: 1.5, calculating UPE around the target site (target accessibility analysis): 17 nt upstream and 13 nt downstream. Afterwards, gene ontology (GO) analysis was performed using R package topGO (version 2.40.0) [83] on targets of differentially expressed miRNAs in order to identify overrepresented/enriched GO terms and significant miRNA targets belonging to enriched GO terms. Classical enrichment analysis was performed with Fisher’s statistical test (*p*-value ≤ 0.05). To obtain a broader picture of the functions of hops miRNA targets, a protein–protein interaction network was constructed using Cytoscape [84]. For targets representing a single node, 10 additional interactors were sought in the string database with a cut-off of 0.8 confidence (score). For each local network cluster comprising the miRNA targets and its interactors, an enrichment analysis was performed using the built-in stringApp [85].

## 5. Conclusions

Hops have become an increasingly important crop agronomically, mainly due to their use in the brewing industry and, more recently, in the pharmaceutical industry. In our study, we characterized miRNAs in hops and identified differentially expressed miRNAs in the roots of susceptible and resistant hop cultivars 24 h after inoculation with the phytopathogenic fungus *V. nonalfalfae*. We identified 56 known miRNAs belonging to 30 different miRNA families and 43 novel miRNAs. In response to Verticillium inoculation, we identified seven and six differentially expressed miRNAs in the susceptible and resistant hop cultivars, respectively, and 11 differentially expressed miRNAs when comparing the susceptible and resistant hop cultivars. The hop cultivars respond to inoculation by altering the expression of different miRNAs. In silico target analysis revealed a total of 49 transcripts that are regulated by differentially expressed miRNAs. According to the gene ontology enrichment analysis, the targets in the susceptible cultivar are involved in protein retention in ER lumen, vesicle-mediated transport and pigment biosynthetic process, etc. In the resistant cultivar, the targets are involved in the auxin-activated (hormonal) signalling pathway and regulation of DNA-templated transcription. The obtained results suggest that miRNAs may play an important role in response and resistance to Verticillium in the resistant hop cultivar. The underlying mechanism related to the observed resistance of the cultivar Wye Target is likely related to miRNA regulation, through which the cultivar modulates biological processes that initiate the growth, development and de novo formation of roots and their vascular tissues when inoculated with *V. nonalfalfae*, which was not observed in the susceptible hop cultivar. The latter shows differential expression of miRNAs, which regulate genes involved in transcriptional reprogramming, leading to changes in metabolism and resulting in unsuccessful defence and the death of the plant.

## Figures and Tables

**Table 1:** Statistics of small RNA sequencing reads from 12 sRNA libraries. First two letters of sample names denote cultivar; CE, susceptible cultivar Celeia; WT, resistant cultivar Wye Target; Ctrl, control; Inoc, inoculated, and numbers denote biological replicates (1–3).

Sample	Num. of Raw Reads	Mean Length of Raw Reads (bp)	Num. of Reads after Processing	Mean Length of Processed Reads (bp)	Reads Mapped to Hop Genome
CE-Ctrl1	10,041,349	18	4,636,681	20	2,634,528 (56.82%)
CE-Ctrl2	6,109,522	16	2,232,764	20	1,341,656 (60.09%)
CE-Ctrl3	8,146,318	17	3,232,808	20	2,044,935 (63.26%)
CE-Inoc1	8,631,607	16	2,672,954	19	1,575,284 (58.93%)
CE- Inoc2	6,991,916	18	3,466,372	20	2,000,154 (57.70%)
CE- Inoc3	6,723,592	14	1,771,295	18	1,230,008 (69.44%)
WT-Ctrl1	6,223,982	20	3,745,411	22	2,094,410 (55.92%)
WT-Ctrl2	5,222,013	19	2,933,213	21	1,729,720 (58.97%)
WT-Ctrl3	7,737,001	20	4,083,377	22	2,393,417 (58.61%)
WT-Inoc1	10,059,037	18	4,435,862	21	2,559,244 (57.69%)
WT-Inoc2	7,537,943	18	3,698,547	20	2,125,103 (57.46%)
WT-Inoc3	6,930,753	20	3,202,208	22	1,811,863 (56.58%)

**Table 2:** Differentially expressed miRNAs in response to inoculation with *V. nonalfalfae* and their target transcripts in hops.

miRNA	Log_2_FC; adj. *p* ≤ 0.1		Sig. Interaction; *p* ≤ 0.05	Target Transcript (Orthologue ID *)
	DE in CE	DE in WT		
hlu-miR156e–f	NS	−0.65	NS	Squamosa promoter-binding-like protein 15 (W9RJ15; hops transcript: GAAW01048142.1) ^4^, Squamosa promoter-binding-like protein 6 (W9QNN5) ^4^, Squamosa promoter-binding-like protein 12 (W9RS10) ^4^, Squamosa promoter-binding-like protein 7 (W9R3S1) ^4^, Squamosa promoter-binding-like protein 13 (W9QLM6) ^4^, Squamosa promoter-binding protein 1 (W9SN75) ^4^, gag-polypeptide of LTR copia-type (3694.POPTR_0008s08210.1), Protein SCO1 homolog 2 (225117.XP_009378785.1), LOB domain-containing protein (W9SE87)
hlu-miR159c–d	1.28	0.95	NS	Putative anion transporter 3 (W9RGS2) ^1,3^, Transcription factor GAMYB (W9QVM8) ^2,4^, Integrase catalytic domain-containing protein (A0A087HSL5) ^1,2,3,4^, Acetyltransferase At3g50280-like (M5VT21) ^2,4^, Transcription factor MYB51-like (102107.XP_008226955.1), Cytochrome p450 (57918.XP_004291627.1)
hlu-miR160b	NS	1.21	NS	Auxin response factor (A0A061FPV2) ^3,4^, Auxin response factor (W9QUH2) ^3,4^, Auxin response factor (W9S7Q7) ^3,4^
hlu-miR164b	NS	NS	−1.4	NAC domain-containing protein 100 (W9QTW9), NAC domain-containing protein (W9QCM5; hops transcript: GAAW01060518.1)
hlu-miR167a–d	−1.5	NS	1.35	Mediator of RNA polymerase II transcription subunit (102107.XP_008227322.1)
hlu-miR167f	−2.1	NS	1.34	Mediator of RNA polymerase II transcription subunit (102107.XP_008227322.1)
hlu-miR169a–d	NS	NS	−2.2	Nuclear transcription factor Y subunit A-8 (W9QJW4), ATPase (W9WDM0), Nuclear transcription factor Y subunit A-1 (W9RR19), Nuclear transcription factor Y subunit A-10 (W9SK30)
hlu-miR169m–n	NS	NS	−1.95	Nuclear transcription factor Y subunit A-10 (W9SK30), Nuclear transcription factor Y subunit A-1 (W9RR19), TATA-binding protein-associated factor (W9R0N8), Exo84_C domain-containing protein (W9SE15)
hlu-miR171g–h	NS	NS	−1.3	Scarecrow-like protein 22 (102107.XP_008238556.1), Scarecrow-like protein 6-like (57918.XP_004306953.1), GRAS domain-containing protein (A0A251QFM0; hop transcript: GAAW01082848.1), NAD(P)-bd_dom domain-containing protein (M5VYK9)
hlu-miR319c–f	NS	0.85	NS	Transcription factor GAMYB (W9QVM8) ^2,4^, Teosinte branched 1, putative isoform 1 (A0A061GDP3) ^4^
hlu-miR390a	NS	NS	−2.2	Regulation of response to stimulus (218851.Aquca_1504_00001.1) ARM repeat superfamily protein isoform 1 (A0A061G6D5) Mitochondrial protein (3827.XP_004514187.1) Cation-transporting ATPase (W9RQ63)
hlu-miR408a–b	NS	−1.41	−3.9	Long chain acyl-CoA synthetase 8 (W9QFE0) ^4^
hlu-miR828a–b	−3.50	NS	2.5	Serine/threonine-protein phosphatase (W9QNS5) ^2^, Nuclear pore membrane glycoprotein (W9S8T7), RNA pol II transcription regulator recruiting activity-*ATMYB5* (3649.evm.model.supercontig_96.57), 3-hydroxyisobutyryl-CoA hydrolase (A0A087GEK8) ^1^
miRNA-363 miRNA-1427	−2.61	NS	2.4	Polyphenol oxidase (W9S222) ^1,2^, ER lumen retaining receptor family (F6HCQ4) ^1,2^, Dynamin-related protein 4C (W9QQY9) ^2^
miRNA-898 miRNA-2452	−2.49	NS	NS	L-threonine ammonia-lyase activity (161934.XP_010694863.1), gag-polypeptide of LTR copia-type (3750.XP_008361163.1), Vacuolar protein sorting-associated protein 54 (W9SA63) ^1^
miRNA-617	NS	NS	−6.35	Wall-associated receptor kinase (F6GSN9), DIS3-like exonuclease 2 (W9QVR2)

Sig. interaction is the difference between the condition effect for the resistant cultivar compared to the condition effect for the susceptible cultivar. * Orthologue ID is either UniProt (Entry) or STRING identifier. NS = not significant. ^1,2^ Significant genes identified in topGO analysis of biological process and molecular function ontologies, respectively, performed with targets of differentially expressed miRNAs in the susceptible cultivar. ^3,4^ Significant genes identified in topGO analysis of a biological process and molecular function ontologies, respectively, performed with targets of differentially expressed miRNAs in the resistant cultivar.

## Data Availability

The datasets generated and analysed during the current study are available in the NCBI Sequence Read Archive (SRA) repository (https://www.ncbi.nlm.nih.gov/sra/; accessed on 30 August 2021) under the BioProject accession PRJNA665133 (https://www.ncbi.nlm.nih.gov/bioproject/PRJNA665133; accessed on 30 August 2021) and SRA accession numbers; SRR12696058, SRR12696057, SRR12696054, SRR12696053, SRR12696052, SRR12696051, SRR12696050, SRR12696049, SRR12696048, SRR12696047, SRR12696056, SRR12696055. The hop draft genome was obtained from the HopBase genomic resource which is available at http://hopbase.org (accessed on 30 August 2021) and http://hopbase.cgrb.oregonstate.edu (accessed on 30 August 2021) [30,31]. The computationally annotated hop transcriptome is available from our laboratory and the raw NGS sequences are publicly available at NCBI’s SRA archive under BioProject number PRJNA342762, BioSample SAMN05767836, SRA run SRR4242068: https://www.ncbi.nlm.nih.gov/sra/?term=SRR4242068 (accessed on 30 August 2021) [82].

## Supplementary Materials

The following are available on-line at www.mdpi.com/article/10.3390/plants10091883/s1, Table S1: Known miRNAs identified in *Humulus lupulus*. miRNAs were predicted in hop draft genome sequences obtained from HopBase using the miRNA prediction pipeline (miR-PREFeR), followed by alignment of predicted mature miRNA sequences with known miRNAs deposited in miRBase; Table S2: Novel miRNAs identified in *Humulus lupulus*. miRNAs were predicted in hop draft genome sequences obtained from HopBase using the miRNA prediction pipeline (miR-PREFeR); Table S3: Results of differential expression analysis of miRNAs in roots of the susceptible cultivar Celeia performed by DESeq2; Table S4: Results of differential expression analysis of miRNAs in roots of the resistant cultivar Wye Target performed by DESeq2; Table S5: Results of differential expression analysis of miRNAs between root samples of the susceptible cultivar Celeia and the resistant cultivar Wye Target in response to inoculation with *Verticillium nonalfalfae*; Table S6: Enriched GO terms of genes targeted by identified differentially miRNAs in the susceptible hop cultivar Celeia. GO analysis was performed with topGO; Table S7: Enriched GO terms of genes targeted by identified differentially miRNAs in the resistant hop cultivar Wye Target. GO analysis was performed with topGO; Table S8: Differentially expressed miRNAs in response to inoculation with *V. nonalfalfae* and their target transcripts in hops. The cleavage sites within the hop transcripts were predicted on-line using psRNATarget analysis server (2017 Update).
